# Disparities in multiple chronic conditions within populations

**DOI:** 10.15256/joc.2013.3.24

**Published:** 2013-12-24

**Authors:** Efrat Shadmi

**Affiliations:** ^1^Faculty of Social Welfare and Health Sciences, Department of Nursing, University of Haifa, Israel

**Keywords:** Disparities, minority groups, social, multimorbidity, multiple chronic conditions

## Abstract

Disadvantaged populations are disproportionately affected by multiple chronic conditions (MCCs), yet few studies examine the prevalence, outcomes, or effectiveness of MCC interventions in minority and socioeconomically deprived individuals and populations. An important first step in understanding MCCs, not only in such diverse population groups, but also in the general population as a whole, is to broaden the definition and scope of MCC measurement, to encompass more than the simple additive effect of clinical conditions, and to include a wide range of health and health-related aspects that interact and make up the full spectrum of multimorbidity. Only with the use of a comprehensive MCC measurement can some of the differences between the disadvantaged populations be adequately detected. Better understanding of the disparities in access to high quality health and healthcare for persons with MCCs can help guide policy and practice aimed at the prevention and amelioration of the effects of MCCs among disadvantaged groups. Indeed, disparity in MCC populations has been identified as a key goal of the U.S. Department of Health and Human Services’ Strategic Framework on MCCs. The aim of the present paper is to describe current knowledge on disparities in the population of persons with MCCs and to guide efforts for the prevention and management of MCCs in disadvantaged populations.

Journal of Comorbidity 2013;3:45–50

## Introduction

The high prevalence of multiple chronic conditions (MCCs) in general, and especially within disadvantaged populations, is widely recognized. Worldwide, minority populations and persons from low socioeconomic backgrounds have greater health needs [1–5], with demographic, economic, social, and cultural challenges to preventing disease manifestation [6], maintaining healthy life styles [7] and accessing high-quality healthcare [8–10]. It is also increasingly acknowledged that care for persons with MCCs requires a patient goal orientation, focused on maximizing the health goals of individual patients with unique sets of multiple risks, conditions, and priorities, rather than an individual disease-centric approach [11]. Disparities in healthcare, defined as the difference in treatment or access not justified by the differences in health status, sociodemographic attributes, or preferences of individuals or populations [12], have long been shown to affect outcomes of various diseases. Current research, however, does not provide insight into how special circumstances of persons with MCCs, especially those from diverse social, economic, and cultural backgrounds, affect the manifestation and management of MCCs. This paper presents the current knowledge and suggests the next steps for advancing research and practice aimed at the prevention and care of disadvantaged populations with MCCs, with special reference to the U.S. Department of Human and Health Services’ (HHS) Strategic Framework on MCCs.

## Preventing MCCs

A plethora of research exists on the identification of risk factors and the prevention of chronic conditions. However, few studies specifically focus on the prevention of MCCs. Both food insufficiency [13] and excess caloric consumption [14, 15] have been shown to be associated with a higher risk of multimorbidity. Additionally, low levels of physical activity have been linked to MCCs [16]. Persons from disadvantaged populations are at increased risk of food insufficiency, as well as obesity and lack of physical activity. Moreover, clustering of physical and psychosocial determinants within disadvantaged populations can catalyze the emergence and progression of MCCs. A recent study of a cohort of earthquake survivors in Armenia showed that, apart from a high body mass index, several psychosocial factors, including perceived low affordability of healthcare services, poor living standards, lower education, stressful life events, and poor social support, were predictors of incident multimorbidity [17]. Importantly, the strong significant relationship between age and multimorbidity was, in fact, largely attributed to the number of stressful life events, suggesting that experiencing such stressful events during one’s lifespan might be a greater risk factor for incident multimorbidity than the lifespan itself.

As many chronic conditions share similar risk factors, namely, tobacco use, alcohol abuse, an unhealthy diet, and physical inactivity [18], lessons for the prevention of MCCs can be drawn from the general literature on chronic disease prevention and disparity reduction. A major challenge to achieving success in chronic disease prevention is that it requires large-scale policy changes and investment at the population level. However, once implemented, unlike treatment that is often focused on a single disease, most of these actions can potentially prevent the occurrence of multiple conditions, and therefore have very large benefits for the health of populations [19].

Smoking, for example, is one of the most well-established risk factors for lung and other cancers, cardiovascular diseases, obstructive lung disease, and many other chronic conditions [20]. Some smoking-cessation interventions hold the risk of increasing the tobacco-use gap between low- and high-income populations, such as non-subsidized nicotine replacement therapy, which is more readily available and affordable for high-income populations [21]. Yet, other preventive strategies hold promise for disparity reduction. For example, interventions, such as bans on smoking in certain locations, universally affect a wide range of populations, or tobacco taxation, which disproportionately affects the poorest [22].

## Managing MCCs

Managing MCCs requires integration of several self-care tasks for complex treatment regimes of potentially interacting conditions [23]. Minority patients, and especially those with low health literacy, limited education, and/or poor linguistic proficiency, may be especially prone to significant barriers to successful management of their condition [24], particularly when faced with multiple self-care requirements. Managing MCCs also requires coordination amongst the various treatment recommendations from multiple providers [25], ensuring that appointments are not missed and effectively navigating the healthcare system. For persons from disadvantaged populations, this may be particularly demanding [26, 27], due to the multitude of personal, financial, and organizational barriers to integrated care.

To date, however, there is little knowledge on how the specific care management needs of low socioeconomic and minority populations with MCCs can be met. Social support, for example, which is generally viewed as an important resource for self-care of MCCs [23], was not strongly related to self-care behaviors in a study of rural Appalachians with MCCs [28]. That study showed that patients with MCCs did not rely heavily on informal support to help them manage their multiple morbidities, preferring to call on their formal providers, or on self-reliance. Similarly, interventions in individuals with MCCs may have a differential impact in different populations. For example, a multisite, prospective, randomized, controlled study of diabetes patients with multimorbidity in Glasgow, Scotland, showed that educational interventions that were culturally appropriate could improve diabetes knowledge, attitudes and practice in patients from different ethnic minority groups [29]. The intervention was also effective in improving knowledge and attitudes towards complications in the White control group. However, the differences observed between the ethnic minority and White control groups were not significant. Similarly, a meta-analysis of patient education interventions failed to demonstrate any differential impact among different population groups [30]. In that study, the interventions were effective in increasing physical activity among adults with diverse (usually multiple) chronic illnesses, but they were unrelated to gender, age, ethnicity, and socioeconomic distribution [30]. Overall, these findings have important implications for the development of self-management support programs in diverse population groups.

A core feature of health systems that has been shown to reduce disparities is the provision of primary care services [31]. Both primary care supply and attributes: first-contact access for each need; person (rather than disease) focused care; comprehensive care for most health needs; and coordinated care, have been shown to contribute to a more equitable distribution of health in populations, a finding that holds in both cross-national and within-national studies [31]. These same attributes are also connected to better care for patients with MCCs. MCC patients can significantly benefit from first-contact access, as their problems are often non-differentiated. Furthermore, person-focused care can help lead to more informed decisions about the patient’s priorities. Importantly, coordination of the care by multiple providers and services with comprehensive integration is imperative [32]. However, further research is needed in order to delineate the specific pathways by which primary care achieves disparity reduction within populations with MCCs.

## Strategic framework on MCCs

The strategic framework on MCCs [33] specifically addresses the need to improve the understanding of the roles of disparities in persons and populations with MCCs, calling for research that will more clearly elucidate differences between, and opportunities for, intervention among various population groups, and using research to leverage disparity reduction programs. As indicated by Parkeh and Goodman in this issue [34], the HHS’s principal research effort on disparities in MCC populations is a study that aims to describe data systems and data sets that can be analyzed to better improve understanding of, and approaches to, addressing disparities in MCC populations. This effort focuses on describing MCC combinations that are most important for targeting interventions.

While there is little knowledge on how co-occurring chronic diseases are distributed across diverse population groups, current research points to several MCC combinations that are prevalent in low socioeconomic and minority populations. A recent Scottish study on the distribution of multimorbidity showed a clear link between socioeconomic deprivation and multimorbidity, particularly multimorbidity that included combinations of mental and physical health disorders but not combinations of physical illnesses alone [2]: the prevalence of both a mental and physical health disorder increased gradually with every income deprivation decile (from 5.9% in the most affluent group to 11.0% in the least affluent group), while the prevalence of co-occurring physical illnesses was similar across most income deprivation deciles. Moreover, that study showed that the combinations of chronic obstructive pulmonary disease, coronary heart disease, diabetes, or cancer with each other, or with stroke, painful conditions, depression or anxiety, were more prevalent in persons living in deprived versus affluent areas. A reverse income gradient was seen for combinations of the above four index conditions and dementia or atrial fibrillation.

Several studies have examined specific combinations of chronic conditions in ethnic minority populations [35–37]. For example, a study of the incidence, prevalence, and mortality of heart attack in Mexican-American elders showed that Mexican patients are more likely to be male, older, and have co-occurring diabetes mellitus, hypertension, and stroke [35]. Other studies showed that ethnic minority patients with diabetes are more likely to have multiple cerbrovascular [36] and cardiovascular [37] risk factors over time compared to non-Hispanic Whites. It should be noted, however, that as with any study on combinations of health conditions, the ability to detect differences in disease combinations is contingent upon the number and types of conditions assessed and the classification of each study as to which disease constitutes the index condition and how comorbidities are defined.

Research, however, needs to move quickly beyond description of existing gaps. Addressing disparities in MCCs warrants a wide-ranging consideration that encompasses the various social determinants and their interactions with MCCs. Several characteristics distinguish the study and implementation of interventions for MCCs in socially disadvantaged populations. For example, age is a major factor. To date, most studies on MCCs focus on the elderly. While MCCs are more prevalent in older adults [2, 38, 39], socially disadvantaged groups are, on average, younger, and, disproportional to their peers, suffer from poorer health [6]. A recent study on the association between MCCs and health-related quality of life (HRQoL) and its variation by socioeconomic deprivation has found that MCCs have a substantial negative impact on HRQoL, which is most severe in areas of deprivation, especially in younger adults [40]. The fact that young adults are markedly predisposed to the negative effects of deprivation on MCCs is an essential area for further investigation, particularly as most current research on MCCs is still focused on older adults [41–43].

Interventions for older persons with MCCs may not be directly transferable to younger, working-age adults. The non-elderly not only have different types of co-occurring conditions than older adults [44], but also different types of social support systems and responsibilities. These challenges can affect patients’ abilities to perform self-care tasks and effectively manage their MCCs [23], especially when adding social and cultural factors, such as unemployment or inadequate language proficiency [45].

Building a strategic framework to improve the health and quality of life of persons with MCCs requires a special focus on disparity reduction that is also due to structural factors. Importantly, where categorical benefits and the type of insurance are generally age-dependent (as is the case in the USA), fostering healthcare changes to improve the health of individuals with MCCs requires a concentrated effort in directing care for non-Medicare-eligible individuals – namely, the socially disadvantaged, younger adults with MCCs. Recent national (USA) and international efforts can help guide these paths [46, 47].

Another important contributor to the need for a special disparity-reduction focus has to do with the nature of program implementation. Research shows that universally applied quality improvement (QI) efforts do not necessarily reduce disparities [48, 49], as socially and economically stable or advantaged populations tend to absorb the interventions earlier, and thus, potentially increasing the care-quality gap. An organization-wide MCC disparity-reduction strategy (including interventions for diabetes, hypertension, and hyperlipidemia control and cancer screening) implemented within Israel’s largest non-profit health plan (Clalit Health Services) provides an example of how to overcome such an “inverse QI law.” Results of implementation of the Clalit disparity-reduction program show that, though historically, improvement in average quality scores was consistently achieved [50], only after specifically directing tailored efforts towards low socioeconomic and minority clinics, did the gaps in health and healthcare subside [51]. Similarly, embarking on any MCC improvement effort, without initially considering the implication for marginalized population groups and the need to provide customized solutions to minority populations, those of low socioeconomic position or anyone otherwise disadvantaged, can potentially increase disparities in MCCs.

## Next steps in MCC disparity research

While it is important to note that a large share of the burden of MCC lies within socially and economically stable populations, several of the initiatives that stem from the MCC framework have important implications for future MCC disparity research and practice:

1. As described by Parkeh and Goodman [34], the FDA is currently examining the question of whether individuals with MCCs are being excluded from controlled clinical trials. To complement this initiative, within a disparity-reduction MCC framework, it is also important to ensure that marginalized groups with MCCs are adequately represented [52].
2. To capture disparities between and within populations, there is a need to broaden the definition and scope of MCC measurement. The MCC framework defines MCCs as two or more concurrent chronic conditions. Defining MCCs as a sum of chronic conditions may have significant implications for the identification of the extent of differences between populations [10], as well as the manifestation and outcomes of MCCs in various population groups. Measures that simply sum chronic diseases may fail to capture the burden associated with multimorbidity [53]. Fried and colleagues, for example, have shown that a substantial amount of the contribution of disease to disability may derive from the interactions of specific chronic diseases, and is well beyond the additive effects of several diseases independently [54]. While such a broad definition of various types of interacting conditions, rather than a count of chronic conditions, is beneficial for any classification of MCCs, for minority or otherwise marginalized populations, a broad definition that captures not only predetermined diagnosed diseases but also acute conditions and general symptoms may be particularly important as disparities in diagnoses exist [55]. Such gaps may result in under-identification of chronic conditions and a need to capture patient complexity based on classification of a wider range of preidentified conditions (i.e. symptoms or risk factors) and their interactions [56, 57].
3. Most MCC literature to date is descriptive or epidemiological in nature [58]. Few studies describe interventions for the treatment of persons with MCCs [59]. Of this limited evidence base, there is minimal consideration of the impact of socioeconomic deprivation; none of the studies that were included in the above-mentioned review considered the possibility of a differential effect of interventions in various socioeconomic groups. Future research should aim to assess MCC interventions in a wide range of population groups, especially considering younger, working-age adults.
4. Healthcare systems and organizations that aim to reduce disparities in prevention and treatment of MCCs should adopt a comprehensive approach, building on both the MCC strategic framework [33] and on well-established principles of disparity reduction. Specifically, systems for documenting population characteristics, such as socioeconomic status, education level, and ethnicity should be established [26]. Such patient and population classification can aid in directing tailored prevention and treatment strategies to those who need them the most. Interventions should be developed based on the understanding that some prevention and treatment approaches can potentially increase disparities within populations with MCCs, particularly as organizational, financial, or health literacy barriers can impede adoption by disadvantaged populations. Healthcare services that provide a strong primary care foundation can significantly improve MCC prevention and management as well as reduce disparities.

## Conclusions

A strategy that focuses on improving the health and healthcare of persons with MCCs is more equitable than a single-disease approach. The priority areas identified by the HHS Strategic Framework – geared towards the general population with MCCs – also have important implications for reducing disparities in MCCs within populations. Focusing on the complete spectrum of morbidity (rather than individual conditions), in which multiple illnesses and health-related needs interact, can more accurately depict the much greater impact of illness among the socially disadvantaged [60]. Meeting the specific objectives of the MCC strategic framework [33], of addressing disparities in MCC populations, should be considered within a broad context of measurement, infrastructure, and intervention. This paper suggests that such an effort should include better documentation of sociocultural characteristics of populations and improved understanding of the role and inter-relatedness of risk factors within distinct population groups; establishment of a strong primary care organization of services; and development of prevention and care-management interventions that are tailored to the intricate needs of populations that experience organizational, economic, and cultural barriers to effective healthcare.

## References

[1] Shi L, Starfield B, Kennedy B, Kawachi I (1999). Income inequality, primary care, and health indicators. J Fam Pract.

[2] Barnett K, Mercer SW, Norbury M, Watt G, Wyke S, Guthrie B (2012). Epidemiology of multimorbidity and implications for health care, research, and medical education: a cross-sectional study. Lancet.

[3] Agborsangaya CB, Lau D, Lahtinen M, Cooke T, Johnson JA (2012). Multimorbidity prevalence and patterns across socioeconomic determinants: a cross-sectional survey. BMC Public Health.

[4] Schafer I, Hansen H, Schon G, Hofels S, Altiner A, Dahlhaus A (2012). The influence of age, gender and socio-economic status on multimorbidity patterns in primary care. First results from the multicare cohort study. BMC Health Serv Res.

[5] Salisbury C, Johnson L, Purdy S, Valderas JM, Montgomery AA (2011). Epidemiology and impact of multimorbidity in primary care: a retrospective cohort study. Br J Gen Pract.

[6] Marmot M, Friel S, Bell R, Houweling TA, Taylor S (2008). Closing the gap in a generation: health equity through action on the social determinants of health. Lancet.

[7] Kurian AK, Cardarelli KM (2007). Racial and ethnic differences in cardiovascular disease risk factors: a systematic review. Ethn Dis.

[8] Schoen C, Doty MM (2004). Inequities in access to medical care in five countries: findings from the 2001 Commonwealth Fund International Health Policy Survey. Health Policy.

[9] Van Doorslaer E, Masseria C, Koolman X (2006). Inequalities in access to medical care by income in developed countries. CMAJ.

[10] Shadmi E, Balicer RD, Kinder K, Abrams C, Weiner JP (2011). Assessing socioeconomic health care utilization inequity in Israel: impact of alternative approaches to morbidity adjustment. BMC Public Health.

[11] Tinetti ME, Fried TR, Boyd CM (2012). Designing health care for the most common chronic condition – multimorbidity. J Am Med Assoc.

[12] Institute of Medicine (2002). Unequal treatment: confronting racial and ethnic disparities in health care.

[13] Sharkey JR (2003). Risk and presence of food insufficiency are associated with low nutrient intakes and multimorbidity among homebound older women who receive home-delivered meals. J Nutr.

[14] Walker AE (2007). Multiple chronic diseases and quality of life: patterns emerging from a large national sample, Australia. Chronic Illn.

[15] Booth HP, Prevost AT, Gulliford MC (2013). Impact of body mass index on prevalence of multimorbidity in primary care: cohort study. Fam Pract.

[16] Autenrieth CS, Kirchberger I, Heier M, Zimmermann AK, Peters A, Döring A (2013). Physical activity is inversely associated with multimorbidity in elderly men: results from the KORA-Age Augsburg Study. Prev Med.

[17] Demirchyan A, Khachadourian V, Armenian HK, Petrosyan V (2013). Short and long term determinants of incident multimorbidity in a cohort of 1988 earthquake survivors in Armenia. Int J Equity Health.

[18] Fine LJ, Philogene GS, Gramling R, Coups EJ, Sinha S (2004). Prevalence of multiple chronic disease risk factors. 2001 National Health Interview Survey. Am J Prev Med.

[19] Ezzati M, Riboli E (2012). Can noncommunicable diseases be prevented?. Lessons from studies of populations and individuals. Science.

[20] Glantz S, Gonzalez M (2012). Effective tobacco control is key to rapid progress in reduction of non-communicable diseases. Lancet.

[21] Bernstein SL, Cabral L, Maantay J, Peprah D, Lounsbury D, Maroko A (2009). Disparities in access to over-the-counter nicotine replacement products in New York City pharmacies. Am J Public Health.

[22] Gaziano TA, Pagidipati N (2013). Scaling up chronic disease prevention interventions in lower- and middle-income countries. Annu Rev Public Health.

[23] Bayliss EA, Ellis JL, Steiner JF (2007). Barriers to self-management and quality-of-life outcomes in seniors with multimorbidities. Ann Fam Med.

[24] Shaw SJ, Armin J, Torres CH, Orzech KM, Vivian J (2012). Chronic disease self-management and health literacy in four ethnic groups. J Health Commun.

[25] Anderson G, Horvath J (2002). Chronic conditions: making the case for ongoing care.

[26] Chin MH, Clarke AR, Nocon RS, Casey AA, Goddu AP, Keesecker NM (2012). A roadmap and best practices for organizations to reduce racial and ethnic disparities in health care. J Gen Intern Med.

[27] Shadmi E (2013). Quality of hospital to community care transitions: the experience of minority patients. Int J Qual Health Care.

[28] Bardach SH, Tarasenko YN, Schoenberg NE (2011). The role of social support in multiple morbidity: self-management among rural residents. J Health Care Poor Underserved.

[29] Baradaran HR, Knill-Jones RP, Wallia S, Rodgers A (2006). A controlled trial of the effectiveness of a diabetes education programme in a multi-ethnic community in Glasgow [ISRCT28317455]. BMC Public Health.

[30] Conn VW, Hafdahl AR, Brown SA, Brown LM (2008). Meta-analysis of patient education interventions to increase physical activity among chronically ill adults. Patient Educ Couns.

[31] Starfield B, Shi L, Macinko J (2005). Contribution of primary care to health systems and health. Milbank Q.

[32] American Geriatrics Society Expert Panel on the Care of Older Adults with Multimorbidity (2012). Patient-centered care for older adults with multiple chronic conditions: a stepwise approach from the American Geriatrics Society: American Geriatrics Society Expert Panel on the Care of Older Adults with Multimorbidity. J Am Geriatr Soc.

[33] U.S. Department of Health & Human Services (2010). Multiple chronic conditions: A strategic framework. Optimum health and quality of life for individuals with multiple chronic conditions.

[34] Parekh AK, Goodman RA (2013). The HHS strategic framework on multiple chronic conditions: genesis and focus on research. J Comorbidity.

[35] Otiniano ME, Ottenbacher KJ, Markides KS, Ray LA, Du XL (2003). Self-reported heart attack in Mexican-American elders: examination of incidence, prevalence, and 7-year mortality. J Am Geriatr Soc.

[36] Towfighi A, Markovic D, Ovbiagele B (2012). Current national patterns of comorbid diabetes among acute ischemic stroke patients. Cerebrovasc Dis.

[37] Egede LE, Gebregziabher M, Lynch CP, Gilbert GE, Echols C (2011). Longitudinal ethnic differences in multiple cardiovascular risk factor control in a cohort of US adults with diabetes. Diabetes Res Clin Pract.

[38] Taylor AW, Price K, Gill TK, Adams R, Pilkington R, Carrangis N (2010). Multimorbidity – not just an older person’s issue. Results from an Australian biomedical study. BMC Public Health.

[39] Fortin M, Bravo G, Hudon C, Vanasse A, Lapointe L (2005). Prevalence of multimorbidity among adults seen in family practice. Ann Fam Med.

[40] Lawson KD, Mercer SW, Wyke S, Grieve E, Guthrie B, Watt B (2013). Double trouble: The impact of multimorbidity and deprivation on preference-weighted health related quality of life a cross sectional analysis of the Scottish Health Survey. Int J Equity Health.

[41] Weiss CO, Boyd CM, Yu Q, Wolff JL, Leff B (2007). Patterns of prevalent major chronic disease among older adults in the United States. J Am Med Assoc.

[42] Marengoni A, Rizzuto D, Wang HX, Winblad B, Fratiglioni L (2009). Patterns of chronic multimorbidity in the elderly population. J Am Geriatr Soc.

[43] Salive ME (2013). Multimorbidity in older adults. Epidemiol Rev.

[44] Boyd CM, Weiss CO, Halter J, Han KC, Ershler WB, Fried LP (2007). Framework for evaluating disease severity measures in older adults with comorbidity. J Gerontol A Biol Sci Med Sci.

[45] O’Brien R, Wyke S, Guthrie B, Watt G, Mercer S (2011). An ‘endless struggle’: a qualitative study of general practitioners’ and practice nurses’ experiences of managing multimorbidity in socio-economically deprived areas of Scotland. Chronic Illn.

[46] Boyd C, Leff B, Weiss C, Wolff J, Clark R, Richards T (2010). Clarifying multimorbidity to improve targeting and delivery of clinical services for Medicaid populations. Faces of Medicaid Data Series. Center for Health Care Strategies, Inc.

[47] Freund T, Peters-Klimm F, Rochon J, Mahler C, Gensichen J, Erler A (2011). Primary care practice-based care management for chronically ill patients (PraCMan): study protocol for a cluster randomized controlled trial [ISRCTN56104508]. Trials.

[48] Trivedi AN, Zaslavsky AM, Schneider EC, Ayanian JZ (2005). Trends in the quality of care and racial disparities in Medicare managed care. N Engl J Med.

[49] Sequist TD, Adams A, Zhang F, Ross-Degnan D, Ayanian JZ (2006). Effect of quality improvement on racial disparities in diabetes care. Arch Intern Med.

[50] Rosen B, Porath A, Pawlson LG, Chassin MR, Benbassat J (2011). Adherence to standards of care by health maintenance organizations in Israel and the USA. Int J Qual Health Care.

[51] Balicer RD, Shadmi E, Lieberman N, Greenberg-Dotan S, Goldfracht M, Jana L (2011). Reducing health disparities: strategy planning and implementation in Israel’s largest health care organization. Health Serv Res.

[52] Furler J, Magin P, Pirotta M, van Driel M (2012). Participant demographics reported in “Table 1” of randomised controlled trials: a case of “inverse evidence”?. Int J Equity Health.

[53] Fortin M, Bravo G, Hudon C, Lapointe L, Dubois MF, Almirall J (2006). Psychological distress and multimorbidity in primary care. Ann Fam Med.

[54] Fried LP, Bandeen-Roche K, Kasper JD, Guralnik JM (1999). Association of comorbidity with disability in older women: the Women’s Health and Aging Study. J Clin Epidemiol.

[55] Fiscella K, Holt K, Meldrum S, Franks P (2006). Disparities in preventive procedures: comparisons of self-report and Medicare claims data. BMC Health Serv Res.

[56] Valderas JM, Starfield B, Sibbald B, Salisbury C, Roland M (2009). Defining comorbidity: implications for understanding health and health services. Ann Fam Med.

[57] Huntley AL, Johnson R, Purdy S, Valderas JM, Salisbury C (2012). Measures of multimorbidity and morbidity burden for use in primary care and community settings: a systematic review and guide. Ann Fam Med.

[58] Boyd CM, Fortin M (2010). Future of multimorbidity research: how should understanding of multimorbidity inform health system design?. Public Health Rev.

[59] Smith SM, Soubhi H, Fortin M, Hudon C, O’Dowd T (2012). Managing patients with multimorbidity: systematic review of interventions in primary care and community settings. BMJ.

[60] Starfield B (2011). The hidden inequity in health care. Int J Equity Health.

